# iStent insertion orientation and impact on trabecular meshwork motion resolved by optical coherence tomography imaging

**DOI:** 10.1117/1.JBO.29.7.076008

**Published:** 2024-07-27

**Authors:** Zhaoyu Gong, Murray A. Johnstone, Ruikang K. Wang

**Affiliations:** a University of Washington, Department of Bioengineering, Seattle, Washington, United States; b University of Washington, Department of Ophthalmology, Seattle, Washington, United States

**Keywords:** glaucoma, microinvasive glaucoma surgery, iStent, trabecular meshwork, phase-sensitive optical coherence tomography

## Abstract

**Significance:**

The iStent is a popular device designed for glaucoma treatment, functioning by creating an artificial fluid pathway in the trabecular meshwork (TM) to drain aqueous humor. The assessment of iStent implantation surgery is clinically important. However, current tools offer limited information.

**Aim:**

We aim to develop innovative assessment strategies for iStent implantation using optical coherence tomography (OCT) to evaluate the position and orientation of the iStent and its biomechanical impact on outflow system dynamics.

**Approach:**

We examined four iStents in the two eyes of a glaucoma patient. Three-dimensional (3D) OCT structural imaging was conducted for each iStent, and a semi-automated algorithm was developed for iStent segmentation and visualization, allowing precise measurement of position and orientation. In addition, phase-sensitive OCT (PhS-OCT) imaging was introduced to measure the biomechanical impact of the iStent on the outflow system quantified by cumulative displacement (CDisp) of pulse-dependent trabecular TM motion.

**Results:**

The 3D structural image processed by our algorithm definitively resolved the position and orientation of the iStent in the anterior segment, revealing substantial variations in relevant parameters. PhS-OCT imaging demonstrated significantly higher CDisp in the regions between two iStents compared to locations distant from the iStents in both OD (p=0.0075) and OS (p=0.0437).

**Conclusions:**

Our proposed structural imaging technique improved the characterization of the iStent’s placement. The imaging results revealed inherent challenges in achieving precise control of iStent insertion. Furthermore, PhS-OCT imaging unveiled potential biomechanical alterations induced by the iStent. This unique methodology shows potential as a valuable clinical tool for evaluating iStent implantation.

## 1 Introduction

Glaucoma is a leading cause of irreversible vision impairment worldwide,^1^ marked by optic neuropathy and visual field defects. Elevated intraocular pressure (IOP) is the predominant risk factor contributing to glaucoma.^2^ In a normal eye, aqueous humor primarily drains through the trabecular meshwork (TM), enters Schlemm’s canal (SC), and eventually exits to the venous system.^3^ However, in certain glaucoma eyes, this pathway becomes dysfunctional, resulting in increased resistance to outflow and subsequent elevation of IOP. The elevated IOP produces compressive stress on the optic nerve, which can lead to nerve damage and visual field loss.

**Fig. 1:**
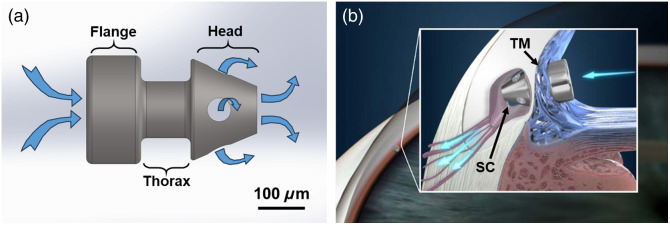
Schematics of the iStent. (a) The diagram of the iStent. Blue arrows illustrate the entrance and exit of aqueous humor. (b) The appearance of implanted iStent in the anterior chamber. The flange resides in the anterior chamber, while the thorax was buried in the trabecular meshwork (TM), and the head in Schlemm’s canal (SC).

Many modalities have been developed to treat glaucoma, targeted at lowering IOP to mitigate optic nerve damage. Microinvasive glaucoma surgery (MIGS) has emerged as a popular option due to its merits of minimal trauma, high efficacy in lowering IOP, safety, and rapid recovery.^4^ The iStent is part of a MIGS subset directed at SC and is one of the most widely used and studied.^5^ Figure 1(a) shows an example of the second-generation iStent, which is manufactured from heparin-coated titanium.^6^ The iStent is implanted into SC using a preloaded inserter.^6^ The fluid pathway in the iStent can create a tunnel through the TM that permits unobstructed aqueous flow from the anterior chamber to the SC lumen [see Fig. 1(b)].

The localization of the iStent can be assessed through various imaging modalities.^7^^,^^8^ Gonioscopy is the most commonly employed method due to its convenience and ability to provide a clear view of the iridocorneal angle. However, it is crucial to note that gonioscopy is a contact imaging technique, and its limitations include the inability to detect the iStent below the tissue surface, resulting in a non-detectable rate of ∼15%.^9^^,^^10^

In contrast, optical coherence tomography (OCT) is a non-invasive imaging modality capable of obtaining depth-resolved reflectivity profiles beneath the tissue surface.^11^ OCT structural imaging has been utilized to gather morphological and positional information of the iStent,^12^ as well as to assess SC dilation.^10^ Nevertheless, current structural OCT imaging of the iStent has certain limitations, including the manual segmentation of the iStent and a lack of comprehensive studies of its three-dimensional (3D) orientation.

The effectiveness of the iStent is primarily evaluated by parameters such as IOP control and medical burden reduction. Numerous *ex vivo*^6^^,^^13^ and *in vivo*^14^^–^^17^ studies have demonstrated the effectiveness of the iStent from the aspects of improved outflow facility, IOP reduction, and decreased medical reliance. In addition, some studies have utilized functional OCT to quantify the improvements in retinal perfusion^18^ and the retention of the retinal nerve fiber layer^19^^,^^20^ induced by the iStent. However, these effectiveness assessments have confounding variables involving indirect measurements focusing on the presumed secondary effects of the iStent, and many involved concomitant cataract surgeries. Given that the iStent is designed to enhance aqueous outflow, assessing device-dependent biomechanical changes in the outflow system could serve as a more direct indicator of iStent effectiveness in altering parameters associated with normalizing aqueous flow and optimizing IOP control.

TM motion stands out as a crucial biomechanical property in the outflow system. During each cardiac cycle, the ocular pulse causes the TM to undergo movement into SC, compressing the SC volume and leading to the discharge of aqueous from SC into the venous system. The magnitude of the periodic TM deformation, typically within 2  μm, correlates with SC volume changes, essentially representing the outflow rate.^21^ Phase-sensitive OCT (PhS-OCT) has demonstrated its capability to quantify pulsatile TM motion *in vivo* with high sensitivity and repeatability.^22^^–^^24^ However, there is currently no research applying this method to evaluate the iStent. Considering that the iStent can increase SC volume,^10^ the TM may exhibit a greater motion amplitude due to the increased volume of aqueous available to discharge per pulse. TM motion changes following iStent implantation have not previously been studied.

The primary goal of this pilot study was to determine whether PhS-OCT imaging can potentially identify the impact of the iStent on TM motion. Another goal was to determine whether the structural OCT imaging could improve the characterization of the iStent’s placement relative to the TM and SC.

To achieve our goal, we introduced a novel OCT imaging protocol and algorithm aimed at advancing the visualization and assessment of the iStent. First, we improved the iStent structural OCT imaging by developing a semi-automated algorithm to segment the iStent from anterior segment tissue. This segmentation facilitates improved localization and precise measurement of the iStent’s 3D orientation. Moreover, we utilized PhS-OCT to quantitatively assess TM motion in the eyes implanted with the iStent, searching for potential biomechanical motion alterations induced by the iStent. Our methodology in this pilot study is exemplified by imaging of iStents implanted bilaterally in a glaucoma patient’s outflow system.

## 2 Material and Methods

### 2.1 Participant

An 84-year-old male patient diagnosed with normal tension glaucoma was recruited for this pilot study. He had previously undergone bilateral cataract and glaucoma surgery involving four implanted iStents. Before the surgery, IOPs of this patient were right eye (OD), 15 mmHg and left eye (OS), 15 mmHg while using a prostaglandin. Other medications were ineffective or poorly tolerated. More recently, macular edema in both eyes required stopping the prostaglandin, so the patient had been on no glaucoma medications for several months before the current OCT study. Throughout the study, OCT examinations were conducted on the patient on three different days (details in Sec. 2.2). The recorded IOPs on these days for OD/OS were 14.0/12.1, 13.0/11.7, and 15.0/16.0 mmHg. The average IOPs were OD, 14 mmHg and OS, 13.3 mmHg, which were near the preoperative IOP of 15 mm, making it difficult to determine the iStent’s effectiveness in IOP reduction. Nevertheless, the patient no longer relies on a prostaglandin medication, suggesting some benefit. Written informed consent was obtained from the patient before the OCT imaging. The study adhered to the tenets of the Declaration of Helsinki and was approved by the Institutional Review Board of the University of Washington.

### 2.2 OCT Data Acquisition

In this study, we employed a clinical swept-source OCT system (PlexElite, Carl Zeiss Meditec Inc., Dublin, California, United States). The system operates at an A-scan rate of 200 kHz, with a central wavelength of 1050 nm and a spectral bandwidth of 100 nm. The above specifications provide an axial resolution of ∼5  μm in tissue. An adaptor lens developed by Carl Zeiss Meditec was attached to the machine to focus the output beam, enabling anterior segment OCT imaging.

As outlined in Table 1, a series of OCT examination protocols were executed to perform *in vivo* imaging of the iStent.

**Table 1:** Summary of OCT examination.

Scan type	Purpose	Scan pattern	Locations	Repeated scan
3D structural imaging	Localization	$6\times 6\;{\mathrm{mm}}^{2}$ wide field	Corneal limbus circumference	Null
	Measure the orientation	$3\times 1\;{\mathrm{mm}}^{2}$ high definition	At each iStent	
2D phase-sensitive imaging	Quantify the TM motion	3 mm B-scan movie	1. Adjacent to iStent	• The initial day
			2. Between two iStents	• 1 week
			3. Remote	• 2 weeks

**Fig. 2:**
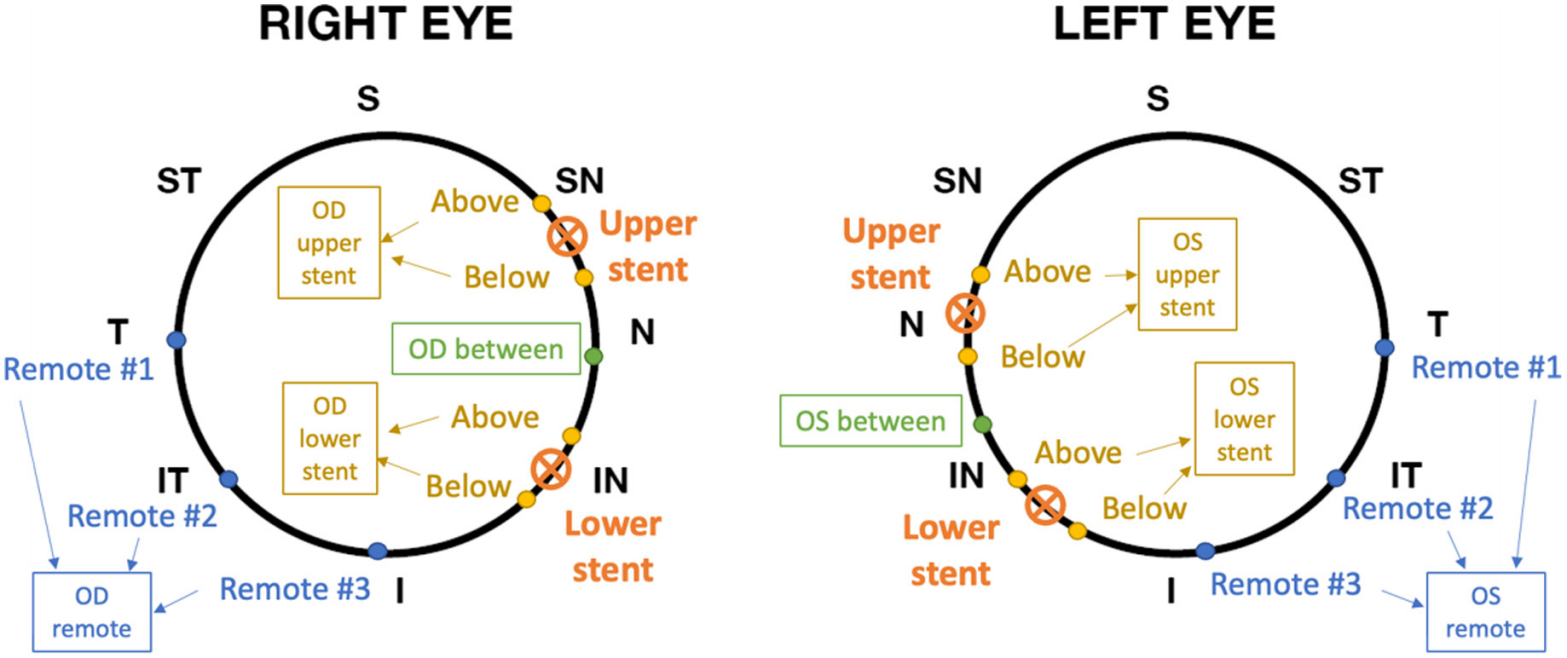
Location map of the iStent and OCT examinations in the eyes of the subject. Orange cross-markers indicate the locations of iStents. Yellow, green, and blue dots indicate the locations examined by PhS-OCT. The arrows and text boxes indicate the location grouping. S, superior quadrant; SN, superonasal quadrant; N, nasal quadrant; IN, inferonasal quadrant; I, inferior quadrant; IT, inferotemporal quadrant; T, temporal quadrant; ST, superotemporal quadrant.

On the initial examination day of the study, both 3D structural imaging and two-dimensional (2D) phase-sensitive imaging were carried out. To begin with, a wide-field scan pattern of $6\times 6\;{\mathrm{mm}}^{2}$ was employed to acquire structural images of the corneal limbus. This scan was repetitively performed around the circumference of both eyes until all four iStents were located and marked (depicted in Fig. 2 by orange marks). Thereafter, a high-definition scan pattern of $3\times 1\;{\mathrm{mm}}^{2}$ was employed to capture the detailed morphology of the iStents. This high-definition pattern consisted of 300 A-scans per B-scan, with a total of 100 B-scan positions. For each B-scan position, we acquired five slightly off-plane repetitions successively and then used the average to reduce the speckle noise. The elongation of the scanning duration due to the repetitions is imperceptible to patients, as the acquisition of each 3D dataset still occurs within 1 s, but the speckle reduction greatly improves the accuracy of iStent segmentation. The iStent was positioned at the center of the scan pattern. In addition, the 3-mm edge of the scan pattern (the fast-scan axis) was aligned perpendicular to the iris circumference, in other words, parallel to the iris radius, by adjusting the rotational angle of the pattern.

Once satisfactory structural images were obtained, 2D phase-sensitive imaging was conducted to quantitatively assess the motion of the TM at various locations. The scan pattern for the phase-sensitive imaging covered a 3-mm linear field and comprised 300 A-scans per B-scan. A total of 3000 B-scans were repetitively captured at the same position (the so-called BM-scan), taking ∼7  s. This scan pattern was also aligned perpendicular to the iris circumference, with the iridocorneal limbus situated at the center of the imaging field. During the scanning process, the cardiac pulse was recorded using a finger clip pulse transducer synchronized with the OCT acquisition. Three distinct types of locations were examined: the region immediately adjacent to the iStent (including both above and below, marked with yellow dots in Fig. 2), the space between two iStents (indicated by green dots in Fig. 2), and remote locations (indicated by blue dots in Fig. 2). For each dot marker in Fig. 2, three repeated measurements were averaged.

Subsequently, the patient returned for two additional phase-sensitive imaging sessions, 1 and 2 weeks after the initial examination, to verify the consistency of the results.

### 2.3 OCT Data Processing

#### 2.3.1 Processing 3D structural images

**Fig. 3:**
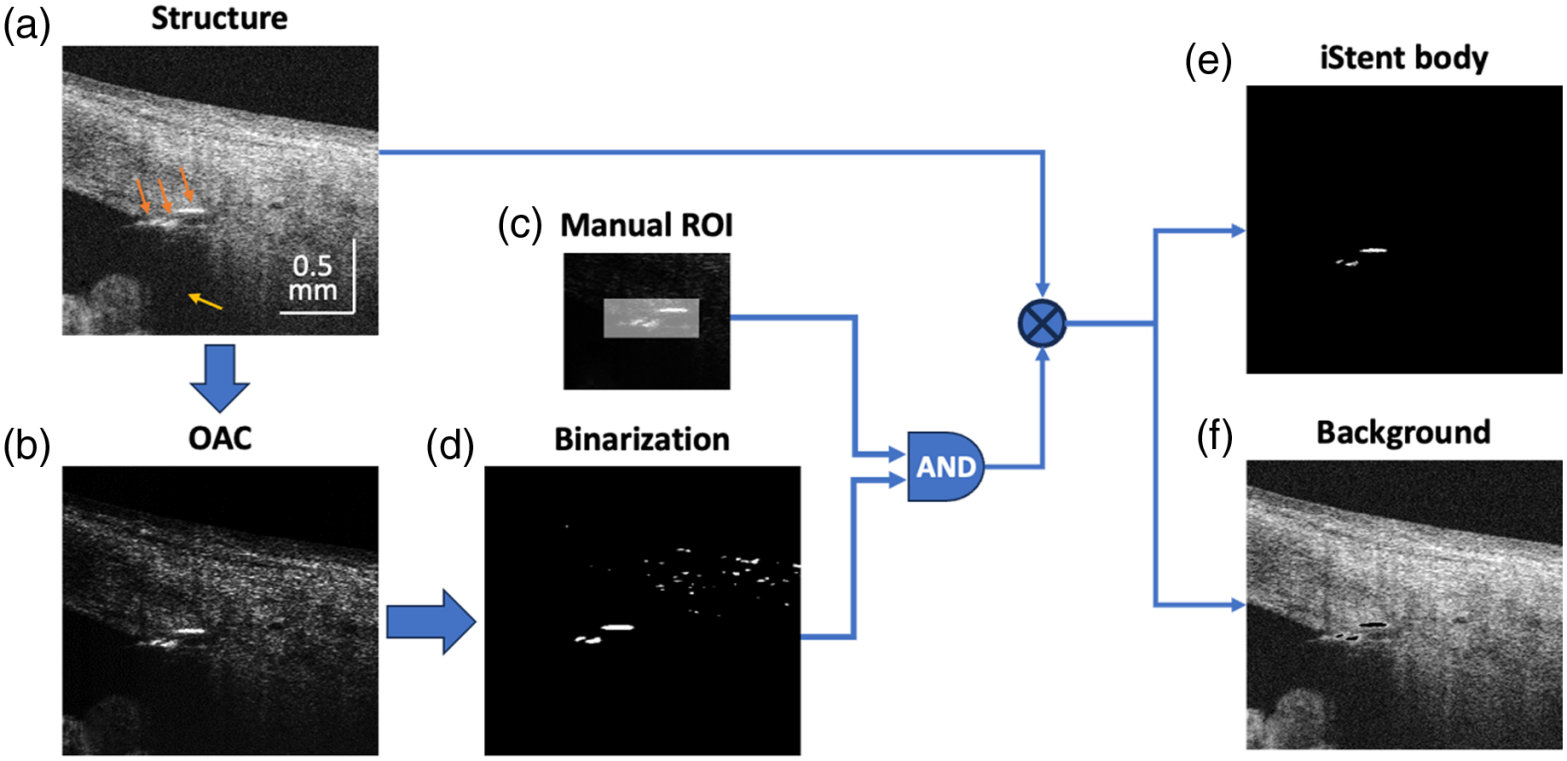
Data processing flowchart of the iStent segmentation. (a) The OCT structural image. Three orange arrows pointed to the head, thorax, and base flange of the iStent, respectively. The yellow arrow indicated the shadow of the iStent. (b) The optical attenuation coefficient (OAC) map. (c) The manually selected region of interest (ROI) for locating iStent. (d) The binarized OAC map. (e) The segmented iStent body. (f) The tissue background.

The purpose of 3D structural imaging is to determine the position and orientation of the iStent within the anterior segment, factors that are crucial for evaluating the success of its implantation. To achieve this goal efficiently and accurately, we developed a semi-automated algorithm to segment the iStent, as illustrated in Fig. 3.

In Fig. 3(a), one OCT cross-sectional image of the iStent was used as an example. On the OCT image, three distinct segments of the iStent can be observed—the head, thorax, and base flange, as indicated by the orange arrows. Note that only the anterior surface of the iStent is visible because the iStent’s metallic body prevents the light from penetrating deeper, leaving a shadowed tail, as indicated by the yellow arrow in Fig. 3(a). Leveraging the attenuation property of the iStent, we employed the optical attenuation coefficient (OAC) to improve the contrast of the iStent with the surrounding tissue. The calculation was based on a depth-resolved OAC algorithm developed for enhancing the segmentation of retinal features in the investigation of age-related macular degeneration.^25^^–^^27^ Thereafter, the iStent mask was generated by combining the binarization of the OAC with a manually selected cubic region of interest (ROI). It is important to note that although the example in Fig. 2 is a cross-section, the algorithm operates on the entire cube. This process effectively isolated the iStent from the surrounding tissue.

**Fig. 4:**
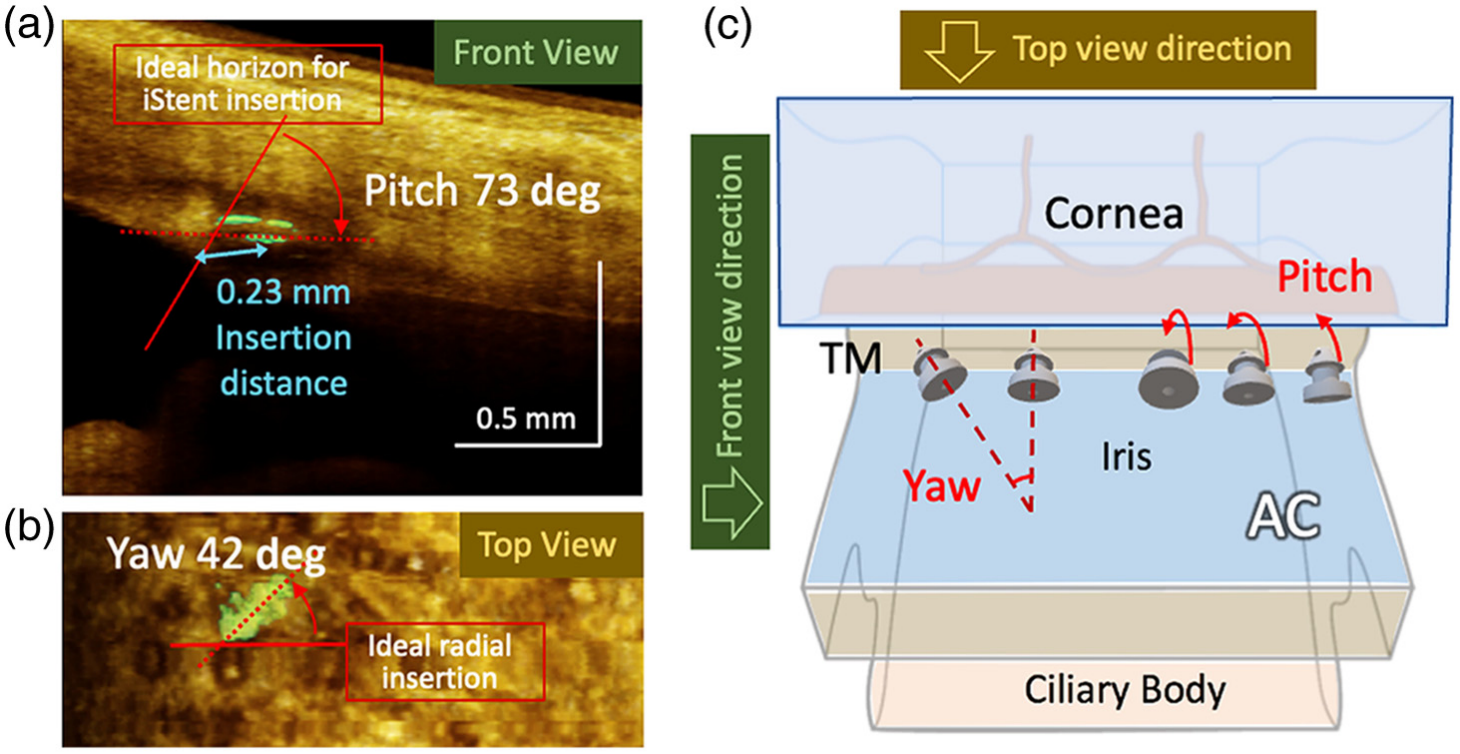
Illustration of iStent position and orientation measurements. (a) The front view of anterior segment 3D rendering. Pitch (red arrow) and insertion distance (blue arrow) extraction are demonstrated. (b) The top view of anterior segment 3D rendering, where yaw extraction is demonstrated. (c) The gonioscopic view of the anterior chamber angle. The concept of yaw and pitch was explained using iStent 3D models, and the directions of the top and front views were illustrated using yellow and green arrows, respectively. TM, trabecular meshwork; AC, anterior chamber. The ideal insertion is orthogonal iStent entry into the TM, minimizing tangential TM tissue disruption. The pitch and yaw are the deviations from their corresponding ideal insertion directions.

Once the segmentation was obtained, a 3D rendering of the anterior segment was created for visualization, with the iStent and the background displayed in different colors. An example is provided in Fig. 4, where Fig. 4(a) is the front view, and Fig. 4(b) is the top view. The projection directions of the front and top views are illustrated in Fig. 4(c). In Figs. 4(a) and 4(b), a series of parameters were extracted to quantify the iStent’s position and orientation:

1. “Insertion distance” was defined as the distance from the middle segment of the iStent to the insertion point on Schwalbe’s line, indicated by the light blue arrow in Fig. 4(a).
2. “Pitch” was defined as the angle between the iStent axis [the red dotted line in Fig. 4(a)] and the ideal insertion [shown as a red solid line in Fig. 4(a)]. An ideal insertion was defined when the iStent was inserted perpendicular to the TM plane; it offers the shortest traveling distance in tissue and results in the least tissue trauma and TM tissue deformation.
3. “Yaw” was defined as the angle between the iStent axis [the red dotted line in Fig. 4(b)] and the ideal radial insertion [shown as a red solid line in Fig. 4(b)]. The ideal radial insertion was defined when the iStent was inserted perpendicular to the iris circumference. Since the OCT fast-scan axis has already been aligned parallel to the iris radius during scanning, the yaw can be directly measured against the horizontal axis of the top view.

A graphic explanation is provided in Fig. 4(c) to illustrate the pitch and yaw. The pitch can be understood as the angle at which the iStent’s head tilts downward from the ideal insertion orientation. The yaw can be understood as the angle that the iStent’s lateral insertion axis rotates away from the ideal radial condition. Any deviation from these ideal insertion conditions involves travel through more tissue with increased trauma and tissue deformation.

#### 2.3.2 Processing 2D phase-sensitive imaging data

**Fig. 5:**
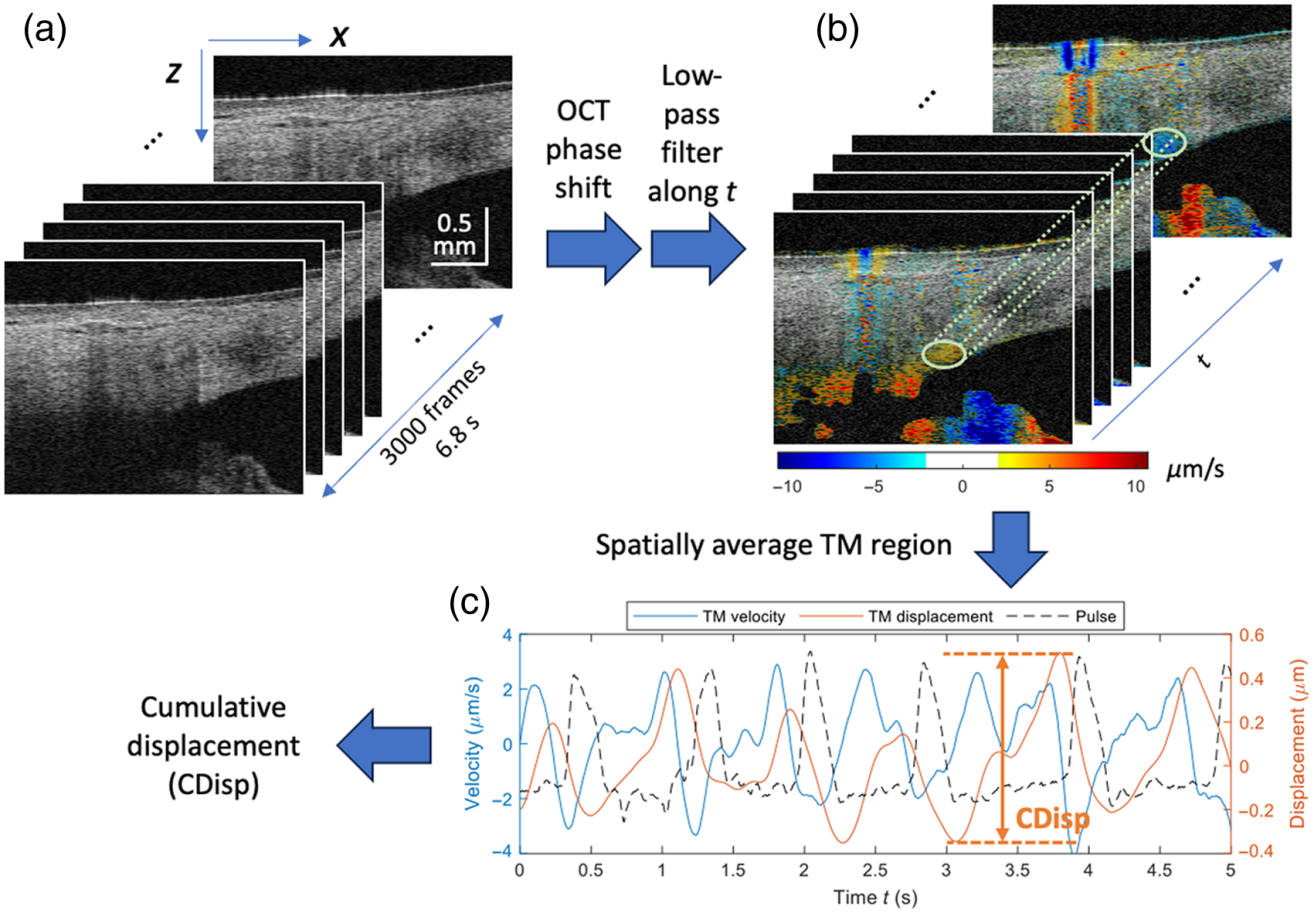
Data processing flowchart of the 2D phase-sensitive image. (a) The stack of 3000 B-scans. (b) The local tissue velocity overlaid on the structural image stack using a pseudo-color encoding. The light green oval indicates the TM region where the velocity waveform was extracted. (c) The waveform of TM velocity, displacement, and the cardiac pulse, which was measured by a finger clip pulse transducer.

The purpose of phase-sensitive imaging is to assess the effectiveness of the iStent by examining its impact on the restoration of TM motion. We used a well-established algorithm to extract the motion of the TM,^22^ as illustrated in Fig. 5. (1) The entire stack of 3000 B-scans was corrected by a subpixel registration algorithm^28^ and a phase compensation algorithm^29^ to minimize bulk eye motion. (2) The phase shift Δφ(x,z,t) between every two adjacent B-scans was extracted and converted into velocity using the Doppler phase formula,^30^ $v(x,z,t)=\frac{\lambda_{0}}{4\pi n\Delta T}\cdot \Delta \varphi (x,z,t)$, where $\lambda_{0}$ is the OCT center wavelength, n is the refractive index of eye tissue, and ΔT is the time elapsed between two adjacent B-scans. For every spatial pixel (x,z), the velocity was denoised along the time axis (t) using a 20-order Butterworth frequency domain low-pass filter with a cutoff frequency of 6 Hz. (3) The pixels within a $50\times 15\;{\mu m}^{2}$ ellipse (with major and minor axes along the x and z axes, respectively) centered at the TM were averaged to create a waveform of TM velocity. The TM displacement was then obtained by the time integral of the velocity.

For evaluation purposes, we used cumulative displacement (CDisp) as the indicator of TM motion strength, which is defined as the TM motion amplitude (the range between the maximum and minimum value of the TM displacement). The CDisp from three independent examination days were compared to verify the consistency of measurements. Moreover, the CDisp from different locations (see Fig. 2, sorted by the relative distance to iStents) were compared to determine whether the iStent had a regional impact on the motion of the TM.

## 3 Results

### 3.1 iStent’s 3D Visualization and Measurement of Position and Orientation

**Fig. 6:**
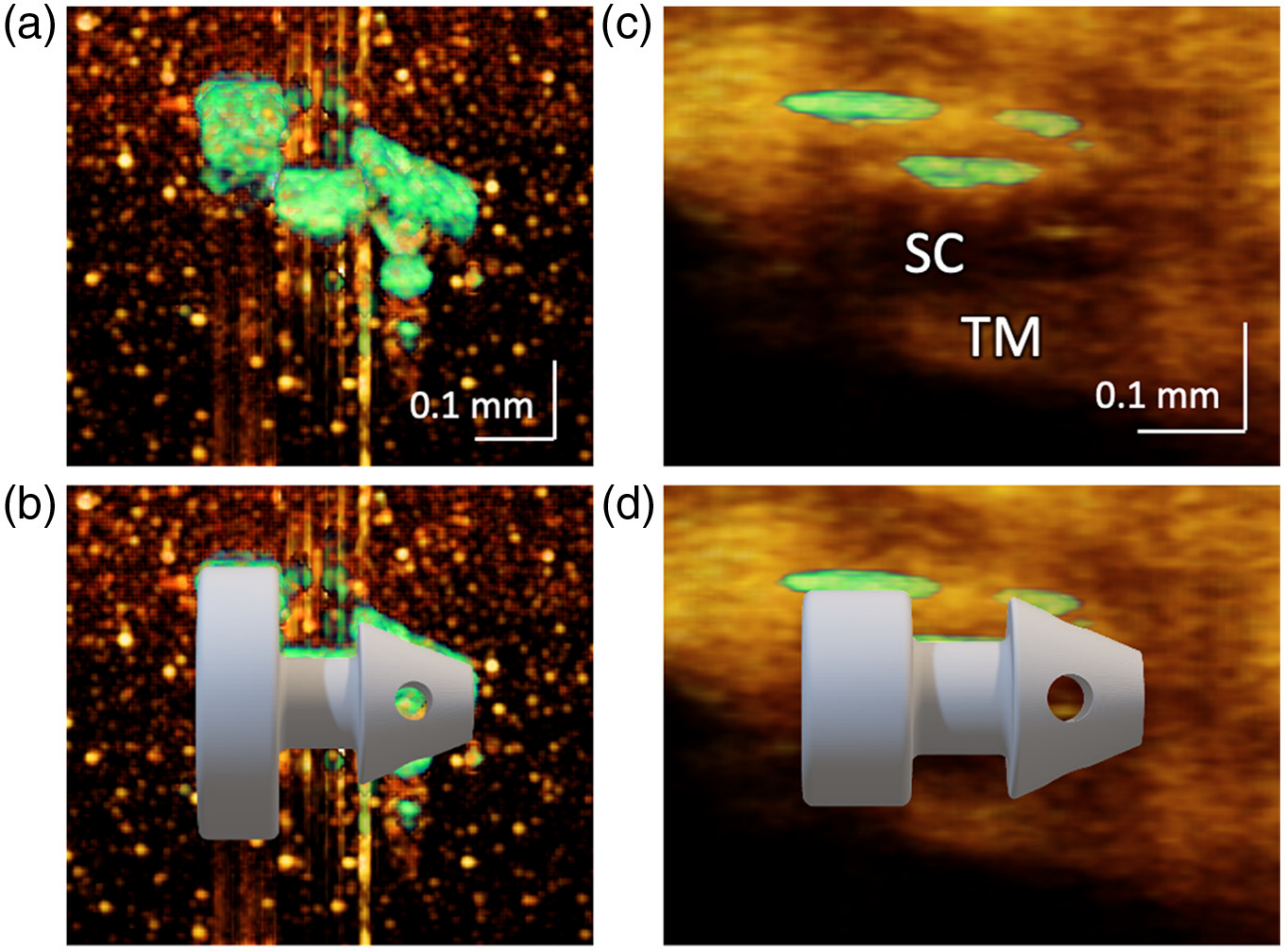
Structural renderings of two different iStent versions. The background was displayed in orange, while the iStent is in green. (a) Image of an iStent-embedded phantom with a wide flange. (b) Overlay of the image with the CAD model. (c) Image of the iStent-implanted eye with the shorter flange. (d) Overlay of the image with the CAD model. The left panel iStent is from the later version, and the right panel iStent is from the earlier iStent version. Both versions align with their respective CAD models.

Figure 6 displays the structural images of two iStent samples. On the left side [Figs. 6(a) and 6(b)], an iStent phantom is displayed, which was built with an iStent embedded in a gelatin substrate. On the right side [Figs. 6(c) and 6(d)], an *in vivo* image of the iStent is displayed. Both were processed and rendered using the algorithm described in Sec. 2.3.1.

Figure 6(a) shows the image of the iStent phantom. Only the surface facing the OCT probe is visible due to the high attenuation of light by the metal. To illustrate the consistency between the image and the object, a computer-aided design (CAD) model, matching the size of the iStent sample, was integrated into the picture. As depicted in Fig. 6(b), the iStent image, displayed in green, aligns well with the model. Figure 6(c) shows the *in vivo* OCT image of the iStent. As can be observed, the *in vivo* image has a lower resolution than the phantom image because of the lower sampling density and the bright tissue background. Despite this fact, three segments were still very distinct and aligned with the CAD model [Fig. 6(d)], which is sufficient for determining the iStent’s position and orientation. Note that the two iStent samples are different versions. The phantom used a newer version (the iStent *inject*^®^ W) with a wider flange, while the one implanted in this patient’s eye was an earlier version (the iStent *inject*^®^) with a smaller flange.

**Fig. 7:**
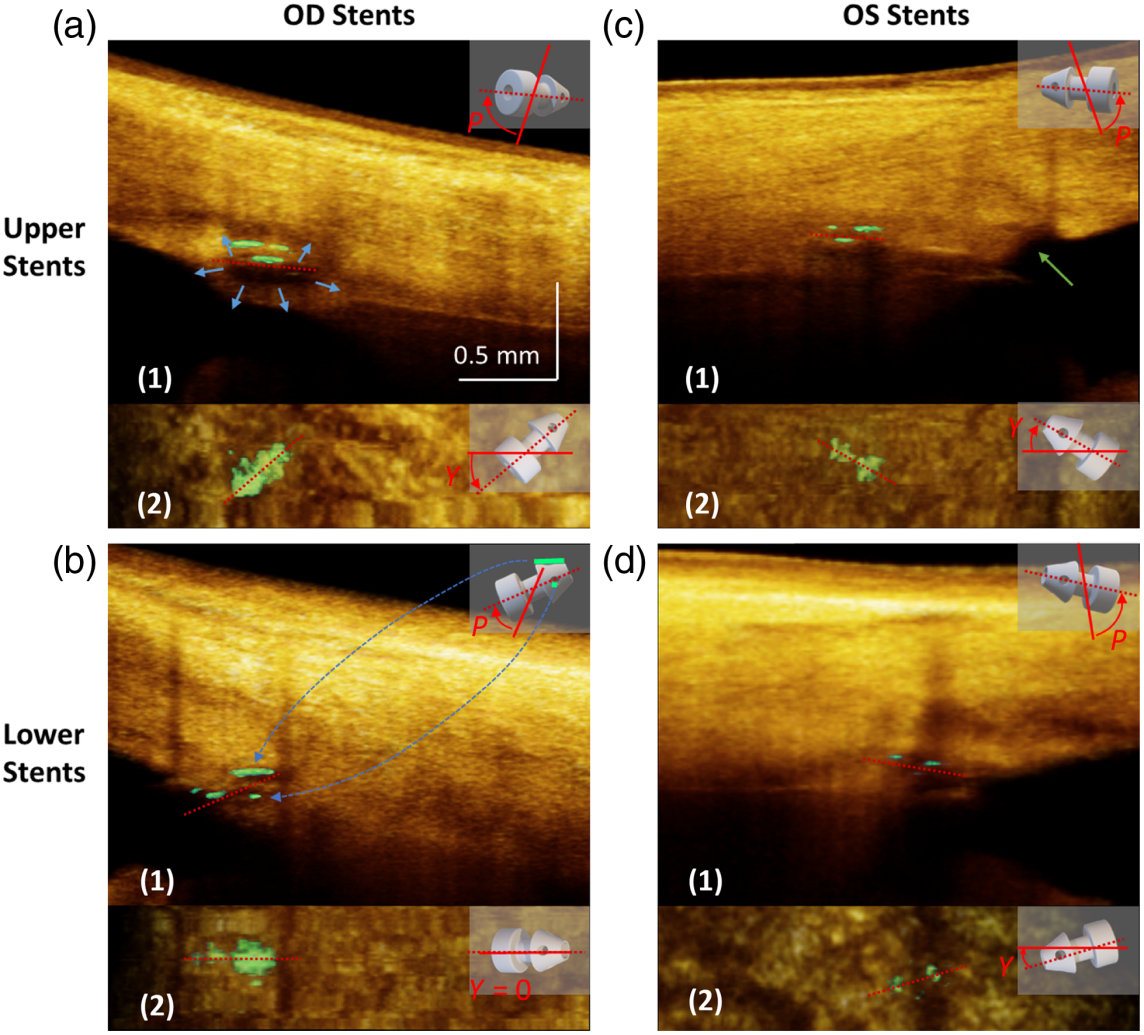
Structural renderings of the four iStents implanted in the patient’s eye. The tissue background is displayed in orange, while the iStent is in green. Structural rendering was displayed using orthographic projection. The front view is obtained by projecting the data cube along the OCT slow-scan axis direction, representing a radial limbal section where the pitch is determined. The top view is obtained by projecting along the OCT A-scan direction, which is parallel to the limbal plane. This orientation permits determining yaw (see Fig. 4 for the explanation of pitch and yaw). In each specific view, the orientation of the iStent axis is indicated using a dotted red line. A CAD model was placed in the upper right corner that integrated both pitch (P) and yaw (Y) of the iStent. The P and Y are also illustrated by the angle between the iStent axis (dotted red line) and the ideal insertion (solid red line, definition refers to Sec. 2.3.1). (a1) Front and (a2) top views of the OD upper stent. The blue arrows indicate the dilation of SC. (b1) Front and (b2) top views of the OD lower stent. Dashed blue arrows show the mapping between the CAD model and the iStent reflection. (c1) Front and (c2) top views of the OS upper stent. The green arrow in panel (c1) shows a disrupted region of the TM anterior to the flange final position associated with an inherent difficulty in controlling the insertion process. (d1) Front and (d2) bottom views of OS lower stent (Video 1, MP4, 3.65 MB [URL: https://doi.org/10.1117/1.JBO.29.7.076008.s1], Video 2, MP4, 3.75 MB [URL: https://doi.org/10.1117/1.JBO.29.7.076008.s2], Video 3, MP4, 5.45 MB [URL: https://doi.org/10.1117/1.JBO.29.7.076008.s3], Video 4, MP4, 4.53 MB [URL: https://doi.org/10.1117/1.JBO.29.7.076008.s4]).

All four implanted iStents underwent imaging and processing to generate 3D renderings. Animated representations showcasing these renderings are available in Videos 1–4, and the orthographic projections are presented in Fig. 7. In the context of orthographic projection, the front view is obtained by projecting along the OCT slow-scan axis direction, while the top view is obtained by projecting along the OCT A-scan direction. To enhance understanding of 3D relationships, a CAD model, aligned with the orientation of each iStent, was situated in the upper-right corner for reference. The extractions of pitch and yaw were also illustrated on the model by displaying the iStent axis (dotted red line), the ideal insertion (solid red line), and the angle between them. Again, the ideal insertions are those minimizing the traveling distance of the iStent in TM tissue, as defined in Sec. 2.3.1.

On the projected views, the insertion distance, pitch, and yaw were measured and recorded in Table 2. The four iStents demonstrate rather sizable variations in position and orientation. Notably, the lower stent in the OD [Fig. 7(b)] appeared to be the shallowest, with the base flange completely in the anterior chamber. In contrast, the upper stent in the OS [Fig. 7(c)] was inserted 0.61 mm deeper into the TM and sclera, causing local disruption of TM tissue [indicated by the green arrow in Fig. 7(c)1]. In addition, as revealed in Table 2, substantial differences were observed in the pitch and yaw angles among all the iStents.

**Table 2:** Summary of the position and orientation measurement of the iStents.

	OD upper stent	OD lower stent	OS upper stent	OS lower stent
Insertion distance	0.23 mm	0.01 mm	0.61 mm	0.25 mm
Pitch	73 deg	51 deg	65.2 deg	67.6 deg
Yaw	42 deg	0 deg	31 deg	18.6 deg
Position grading	2	4	4	3

Apart from the OCT result, the iStent position was also evaluated by gonioscopy, following a recently published grading system.^9^ Grade 1 refers to the most anterior position that inserted in the anterior TM, grade 2 represents the posterior TM insertion, grade 3 represents the scleral spur insertion, and grade 4 represents the most posterior position that inserted in the region below the scleral spur. The grading results are provided in Table 2. Among the four iStents, the OD upper stent is the only grade 2 iStent. Interestingly, it is the only iStent that sufficiently dilated the SC, as indicated by the blue arrows in Fig. 7(a)1. The images of the other three iStents showed no sign of SC dilation.

### 3.2 TM Motion Assessment

**Fig. 8:**
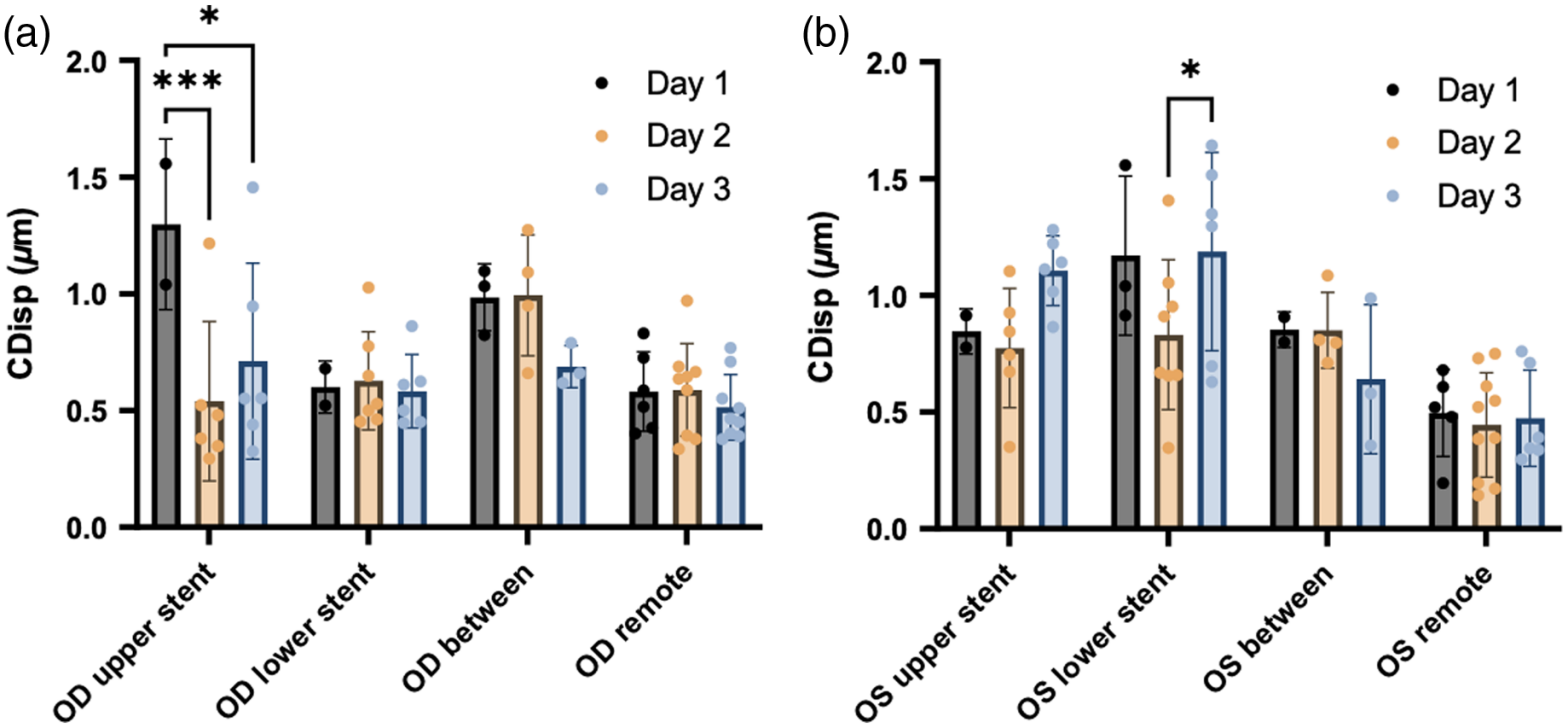
TM motion in (a) OD and (b) OS, binned by the location and day of the measurement. The height of the bar is the average CDisp of all the measurements taken on one specific day and location, and the error bar shows the standard deviation. For the stent groups, the motion from both above and below each specific stent was binned together. For the remote groups, all three remote locations from each eye were binned together. More grouping details can be found in Fig. 2. The results of the pair-wise comparison between different days are displayed in the figure. *p-Value <0.05, **p-value <0.01, and ***p-value <0.001; otherwise, p-value >0.05.

The 2D phase-sensitive imaging data were processed to extract CDisp, following the method described in Sec. 2. First, the CDisp were pregrouped into eight categories according to their positions relative to the iStents (Fig. 8). Eight categories are OD/OS upper stent, OD/OS lower stent, OD/OS between, and OD/OS remote, as shown in arrows and text boxes in Fig. 2. Subsequently, different binning methods were applied to investigate the inter-day variance and the location difference of the TM CDisp.

In the first analysis addressing inter-day variance, CDisp values from different days were separately binned. An inter-day pair-wise comparison using independent samples t-tests was conducted to examine whether the mean value of individual days had statistically significant differences. The results (presented in Fig. 8 by bar plots) indicated that the mean values of the “between” and “remote” groups across 3 days exhibited a compact distribution, demonstrating good reproducibility. Among the stent groups, the OD lower stent and the OS upper stent exhibited closely distributed values. However, the OD upper stent and OS lower stent both displayed 1 day with a significant difference from the other 2 days. Notably, the groups in non-stent areas had better consistency compared to the stent groups.

In the subsequent analysis addressing differences in motion at different circumferential locations, CDisp values from different days were binned together by location. Pair-wise comparisons between days were performed to determine whether individual locations exhibited significantly different CDisp values.

**Fig. 9:**
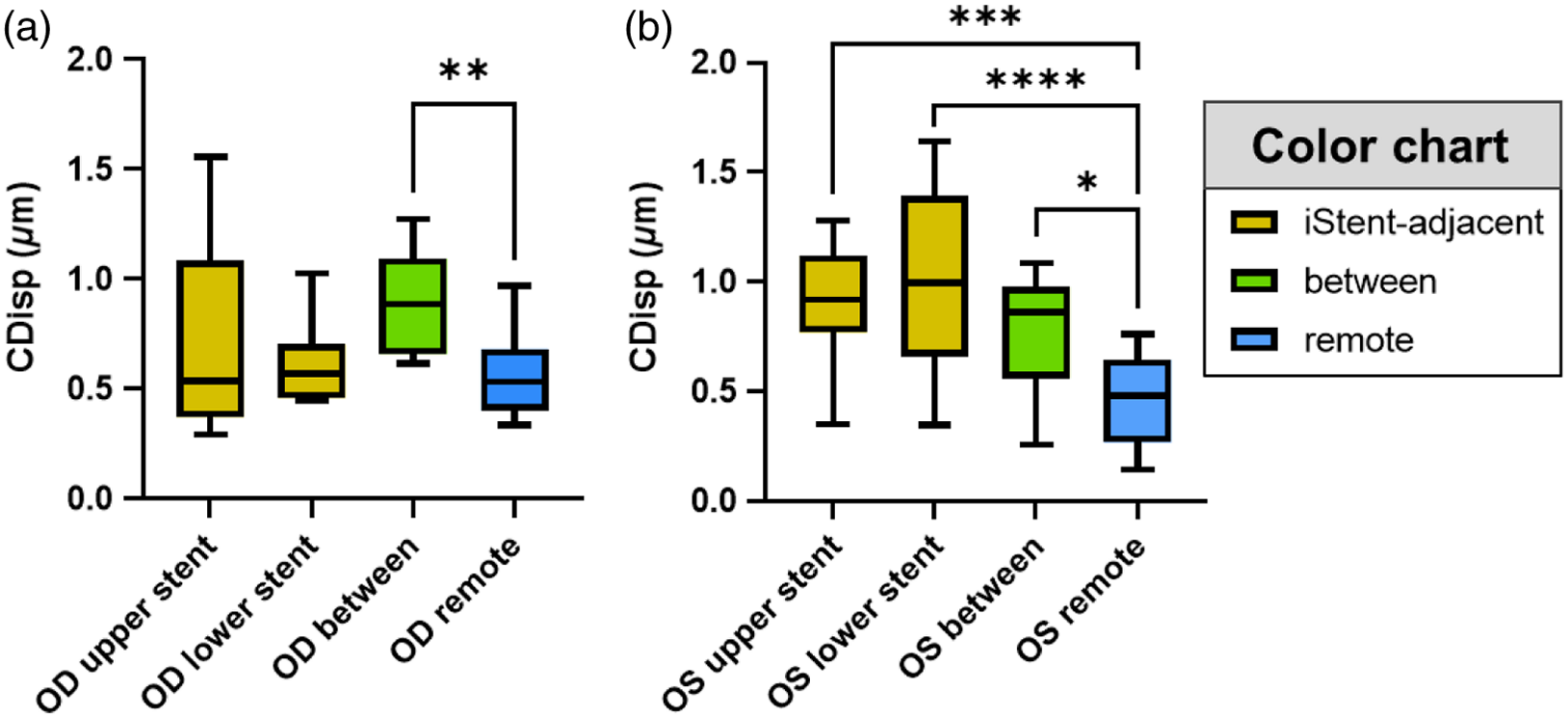
TM motion in (a) OD and (b) OS, binned by locations. The box plot shows the first and third quartiles, and the whiskers show the minimum and maximum. For the stent groups, the motion adjacent to each specific iStent (including both above and below the iStent) measured in all 3 days was binned together and colored yellow. The between-stent groups were colored green. For the remote groups, all three remote locations from each eye measured in all 3 days were binned together and colored blue. More grouping details can be found in Fig. 2. The results of the pair-wise comparison between different days are displayed in the figure. *p-Value <0.05, **p-value <0.01, ***p-value <0.001, and ****p-value <0.0001; otherwise, p-value >0.05.

The results (presented in Fig. 9 by box–whisker plots) revealed that the “between” group showed significantly higher CDisp values than the “remote” group in both OD [Fig. 9(a), p=0.0075] and OS [Fig. 9(b), p=0.0437]. However, stent-adjacent location motion results varied in the two eyes. In the OD upper and lower iStent groups, CDisp values did not show a significant difference when compared to the remote CDisp values. In contrast, the OS upper and lower iStent groups showed highly significant differences.

## 4 Discussion

### 4.1 Insertion Variation Revealed by Structural Imaging

A distinctive innovation of our work was the introduction of a semi-automated iStent segmentation algorithm. Utilizing the highly attenuative property of the iStent’s metallic body to the OCT probing light, we incorporated the OAC conversion of conventional OCT images to enhance the contrast, ensuring efficient and reliable segmentation. The display of the segmented iStent and tissue with different colormaps effectively illustrated the iStent’s position and orientation relative to the anterior segment angle [see Figs. 4(a) and 4(b)]. We introduced three parameters—insertion distance, pitch, and yaw—to quantify the position and orientation of the iStent.

Several noteworthy findings and implications were revealed by the structural imaging. We found that the insertion distance of the device into the TM tends to vary from the manufacturer’s recommendations. According to the manufacturer’s summary,^31^ the head of the iStent should remain in SC, the thorax in TM, and the flange in the anterior chamber. However, only the OD lower stent adhered fully to this recommendation; the other three iStents were inserted more deeply, with portions or all the iStent flange entering TM tissue. Surgeons have a clear view of the anterior chamber angle surface through gonioscopy, but the view behind the flange cannot be seen, limiting the feasibility of accurately assessing the iStent insertion depth during surgery.

**Fig. 10:**
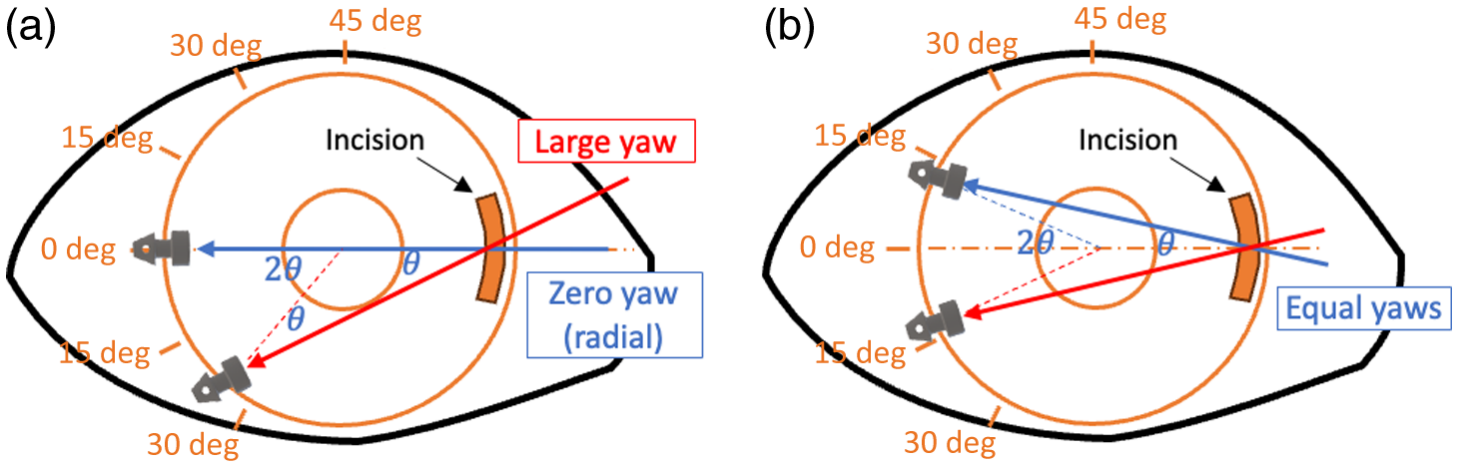
Explanation of yaw variation. (a) The illustration about the ideal radial insertion (blue arrow) and how yaw is affected by the rotation of the insertion device in the iris plane (red arrow). (b) Condition where two iStents have equal yaws. θ denotes the inscribed angle that the two iStents and the incision form; 2θ denotes the central angle that the two iStents form. The degree labels on panels (a) and (b) indicate the calibrations of yaw.

Another finding is that the pitch and yaw angles exhibited considerable variability (see Table 2). Given that the iStent insertion device operates as a long lever, the way it enters the cornea significantly influences the control of the yaw and pitch. For example, the rotation of the device in the iris plane can change yaw, and the yaw is equal to the rotated angle of the device from the ideal radial insertion. To achieve a zero yaw, insertion should occur radially, with the insertion sleeve crossing the iris center, as illustrated in Fig. 10(a) by the blue color. Any deviation from the iris center introduces yaw, as indicated in Fig. 10(b) by the red color. Since the incision location is fixed, yaw must be introduced to at least one of the two iStents. Yaw results in the iStent passing through the TM at an oblique angle, causing more significant TM tissue disruption and deformation than a perpendicular entry. According to the geometric relationship, the summation of the yaws of two iStents equals the inscribed angle that the iStents formed with the corneal incision fulcrum, as marked by θ in Fig. 10(a). A decrease in the yaw of one iStent comes at the cost of an increase in another. A fair strategy to control yaw would be equally distributing two yaw deviations, as illustrated by Fig. 10(b), by placing two iStents symmetrically around the iris diameter crossing the fulcrum. Alternatively, a second paracentesis incision can provide placement at another location without yaw, although this is not part of the standard recommended protocol.

**Fig. 11:**
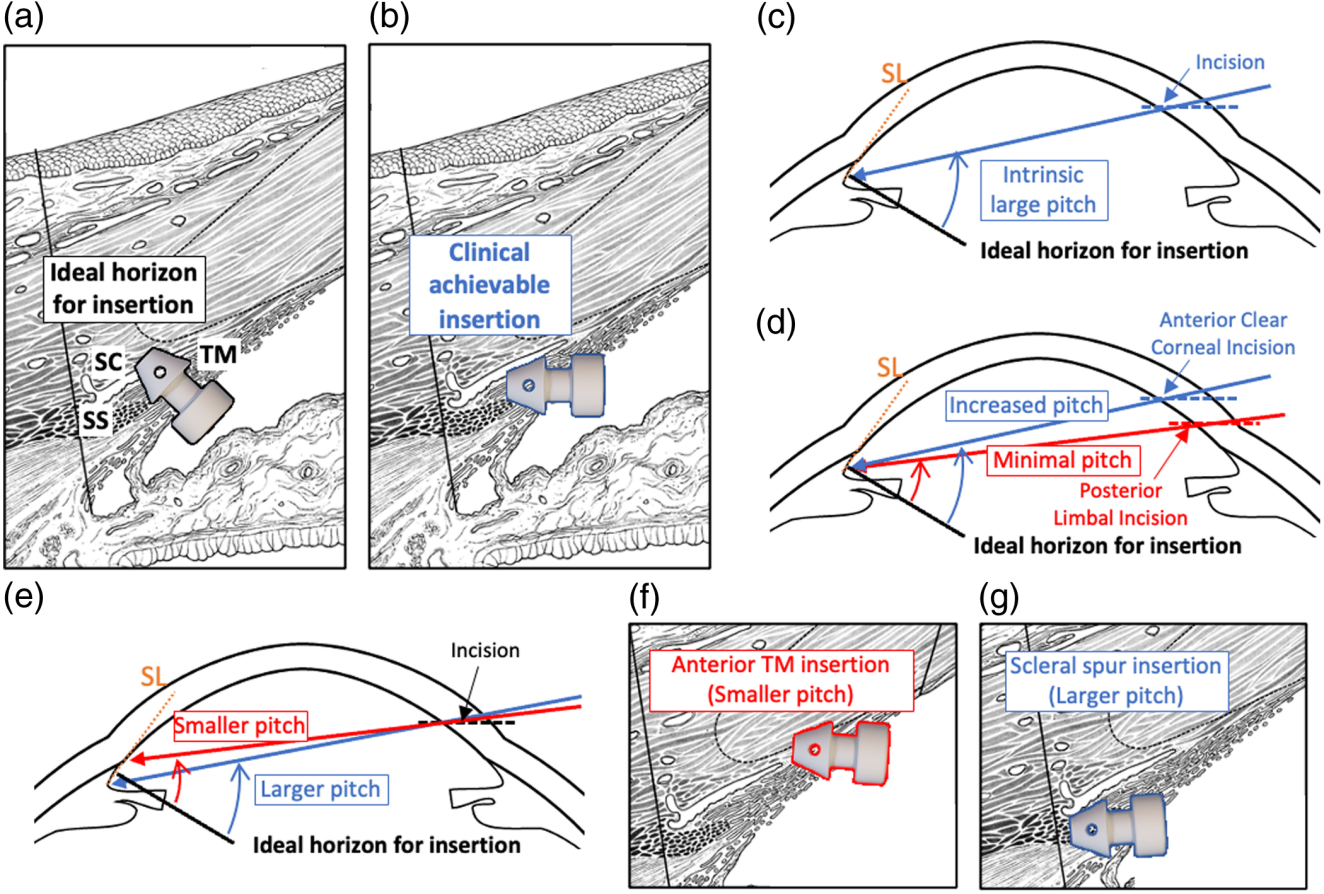
Explanation of pitch variation. (a) The anatomic schematic shows the ideal horizon for insertion, where the iStent faces perpendicular to the TM surface and has minimal travel distance in the TM. (b) The anatomic schematic shows the clinically achievable insertion from the corneal incision. The iStent has a substantially steeper pitch and longer travel distance in TM. (c) The graphical illustration of the anatomic surgical constraint that contributes to the intrinsic large pitch. (d) An illustration of how pitch is affected by the incision location. (e) Illustration of how pitch is affected by the tilting angle of the insertion device. (f) Insertion in the anterior TM results in a smaller pitch but can be anterior to the TM lumen. (g) Scleral spur (SS) level insertion that results in a larger pitch. The orange lines in panels (c)–(e) indicate Schwalbe’s line. The solid black line perpendicular to the TM is the ideal horizon for iStent insertion, which is our reference for measuring pitch. SC, Schlemm’s canal; TM, trabecular meshwork; SS, scleral spur; SL, Schwalbe’s line.

In addition, we observed that all the iStents exhibited a significantly high pitch, exceeding 50 deg. This is attributed to the anatomical constraint that the TM is inclined at an acute angle to the iris plane. In *ex vivo* demonstrations,^6^ iStents can be introduced from the posterior chamber to achieve perpendicular insertion and zero pitch [see Fig. 11(a)]. This effectively minimizes TM trauma as the iStent has the shortest traveling distance in the TM. However, the *in vivo* insertion requires introducing the iStent from an anterior chamber incision [see Fig. 11(b)]. Therefore, an intrinsic large pitch is unavoidable. Figure 11(c) further explains this constraint at a macroscopic scale. As our study shows, the clinically required oblique, somewhat tangential insertion tends to create TM shearing forces, leading to the tearing of TM tissues and posing challenges when effectively targeting SC. This constraint has been affecting other MIGS procedures, such as the femtosecond laser trabeculectomy,^32^ where the laser must enter through a clear cornea, inducing a quite oblique entry into SC. Fortunately, the pitch of iStent placement is minimizable within a limited range in clinical practice, in spite of the anatomical constraint. For example, the incision location is one modifiable factor in determining the pitch. As demonstrated in Fig. 11(d), a minimized pitch can be achieved through a posterior limbal incision, while an anterior clear corneal incision results in a steeper pitch. Furthermore, the tilt of the insertion device around the fulcrum in an anterior–posterior plane can contribute to a surgeon-dependent pitch, as depicted in Fig. 11(e). Close-up views provided in Figs. 11(f) and 11(g) further illustrate the target tissue of iStents under the smaller and larger pitch conditions, respectively. As can be observed in Fig. 11(f), pitch can be minimized by rotating the insertion device anteriorly and targeting the more anterior TM while still positioning over the lumen of SC.

Anatomic studies have demonstrated that the TM anterior–posterior length is ∼700  μm. The lumen of SC is generally localized to the posterior portion of the TM, with the anterior SC lumen limit ∼300 to 350  μm from Schwalbe’s line. The SC length is ∼250 to 350  μm.^33^^,^^34^ iStent placement in the posterior TM is optimal as it can directly enter SC. The OD upper stent imaged in this study corresponds to this condition, as confirmed by the gonioscopy examination. In contrast, inserting into the anterior TM does not ensure entry into the SC lumen [see the example in Fig. 11(f)]. However, favorable deformation of structures surrounding SC may lead to the enlargement of its lumen or that of the distal outflow channels. Moreover, placement at the scleral spur [Fig. 11(g)] or more posteriorly cannot direct the aqueous to the SC lumen either but may improve scleral spur tension and increase aqueous access to the uveoscleral pathways or to the suprachoroidal space.

In essence, our structural imaging technique may help to provide guidance to improve the device placement. Our iStent segmentation algorithm still has much room for improvement. With further optimization, this algorithm has the potential to be integrated into intra-operative iStent OCT imaging. By leveraging deep learning for fully automated iStent segmentation and graphics processing unit acceleration for swift computation, the segmentation and visualization of the iStent can be achieved in milliseconds. This capability has the potential to enable real-time tracking of the iStent intraoperatively using OCT, offering an additional cross-sectional view that cannot be obtained from gonioscopy and leading to more precise implantation.

### 4.2 Biomechanical Impact Revealed by Phase-Sensitive Imaging

Another innovation is the application of phase-sensitive imaging to study the iStent’s effect on TM motion. The technique was previously used to study the biomechanics of the TM by assessing pulse-dependent TM motion, which is reduced in glaucoma patients.^23^ Compared to the current method for assessing the iStent’s effectiveness, which is indirect and involves random, infrequent IOP monitoring, our method measures altered underlying biomechanics that control IOP over time. This ability can serve as a more direct indicator of the iStent’s impact, particularly the impact on factors controlling diurnal pressure.

The results of phase-sensitive imaging reveal two notable findings. First, the TM motion between two iStents consistently exceeded that of remote regions in both OD and OS. A possible explanation is the implanted iStent’s ability to alter the relationships between SC’s walls in adjacent SC areas when the iStent head is in SC. More aqueous can then be directed into SC. The larger space permits greater excursions of the TM in response to the ocular pulse. Greater TM excursions permit a greater stroke volume of aqueous with each cardiac cycle. Moreover, larger SC dimensions reduce resistance to the circumferential flow of aqueous to the site where it enters collector channels. Although some inserted iStents did not enlarge the SC lumen, they disrupted the TM structure [see the green arrow in Fig. 7(c)1], creating an effect similar to a local trabeculectomy, which may still decrease the resistance to aqueous outflow.

Second, the TM motion around different iStents exhibited greater inter-day variability compared to the “between” and “remote” groups, as shown in Fig. 8. A possible reason is the acquisition challenge underlying our OCT scanning protocol. Due to eye motion during the prolonged duration (∼7  s) of 2D phase-sensitive imaging, maintaining the OCT field directly adjacent to the iStent becomes challenging because of the narrow permissible target area. The scanning field centered on the adjacent area frequently drifts closer or farther from the iStent during the imaging process. In contrast, the “between” and “remote” groups were less sensitive to drifting, benefiting from their wider permissible target area.

Interestingly, the TM motion around different iStents behaved differently. As can be seen in Fig. 9, the OS iStent regions have significantly higher CDisp than the OS remote regions [Fig. 9(b)], but the OD stents did not follow this trend [Fig. 9(a)]. This variability may be attributed to the discussed differences in the iStent position, orientation, SC entry success, and the resultant ability to dilate the canal.

An inherent limitation of our study is the use of a single subject. While we observed significantly higher TM motion between two iStents than in the remote region, we cannot definitively attribute this difference to the iStent alone. It could also be due to the intrinsic higher TM motion in the nasal quadrant compared to the temporal quadrant.^24^ To confirm whether the iStent indeed improves TM motion, an ideal approach would involve a longitudinal study comparing pre- and post-operative TM motion or a cross-sectional study comparing TM motion in populations with and without iStents. Despite these limitations, our preliminary results demonstrate the feasibility of evaluating the iStent via its impact on TM motion.

In future work, we will proceed with the suggested longitudinal and cross-sectional studies to further verify the impact of the iStent on TM motion. This subsequent work can significantly enhance understanding of the iStent functional properties and placement requirements and issues crucially important to surgical care and patient outcomes.

## 5 Conclusion

In this pilot study, we introduced a unique OCT imaging protocol and associated algorithms to advance the visualization and assessment of iStent implantation. The 3D OCT structural imaging, combined with the proposed OAC enhancement and segmentation algorithm for iStent, has substantially improved the localization and precise measurement of the iStent’s 3D orientation. The structural imaging results revealed considerable variation regarding the iStent implantation, emphasizing the benefit of achieving a more precise ability to monitor and control this procedure. In addition, the utilization of phase-sensitive imaging provided a new method for evaluating the biomechanical properties of the TM following iStent placement, revealing potential biomechanical alterations induced by the iStent. This comprehensive methodology holds promise as a valuable clinical tool for evaluating iStent implantation and advancing our understanding of the device’s mechanisms for adjusting aqueous outflow.

## Supplementary Material









## Data Availability

Data underlying the results presented in this paper are not publicly available at this time but can be obtained from the authors upon reasonable request.
